# Genetic marker identification of *SEC13* gene for milk production traits in Chinese holstein

**DOI:** 10.3389/fgene.2022.1065096

**Published:** 2023-01-04

**Authors:** Ruike Jia, Lingna Xu, Dongxiao Sun, Bo Han

**Affiliations:** ^1^ Department of Animal Genetics and Breeding, College of Animal Science and Technology, Key Laboratory of Animal Genetics, Breeding and Reproduction of Ministry of Agriculture and Rural Affairs, National Engineering Laboratory for Animal Breeding, China Agricultural University, Beijing, China; ^2^ National Dairy Innovation Center, Hohhot, China

**Keywords:** genetic marker, SNP, milk yield and composition, dairy cattle, dual-luciferase reporter assay

## Abstract

*SEC13* homolog, nuclear pore and COPII coat complex component (*SEC13*) is the core component of the cytoplasmic COPII complex, which mediates material transport from the endoplasmic reticulum to the Golgi complex. Our preliminary work found that *SEC13* gene was differentially expressed in dairy cows during different stages of lactation, and involved in metabolic pathways of milk synthesis such as citric acid cycle, fatty acid, starch and sucrose metabolisms, so we considered that the *SEC13* might be a candidate gene affecting milk production traits. In this study, we detected the polymorphisms of *SEC13* gene and verified their genetic effects on milk yield and composition traits in a Chinese Holstein cow population. By sequencing the whole coding and partial flanking regions of *SEC13*, we found four single nucleotide polymorphisms (SNPs). Subsequent association analysis showed that these four SNPs were significantly associated with milk yield, fat yield, protein yield or protein percentage in the first and second lactations (*p* ≤.0351). We also found that two SNPs in *SEC13* formed one haplotype block by Haploview4.2, and the block was significantly associated with milk yield, fat yield, fat percentage, protein yield or protein percentage (*p* ≤ .0373). In addition, we predicted the effect of SNP on 5′region on transcription factor binding sites (TFBSs), and found that the allele A of 22:g.54362761A>G could bind transcription factors (TFs) GATA5, GATA3, HOXD9, HOXA10, CDX1 and Hoxd13; and further dual-luciferase reporter assay verified that the allele A of this SNP inhibited the fluorescence activity. We speculate that the A allele of 22:g.54362761A>G might inhibit the transcriptional activity of *SEC13* gene by binding the TFs, which may be a cause mutation affecting the formation of milk production traits in dairy cows. In summary, we proved that *SEC13* has a significant genetic effect on milk production traits and the identified significant SNPs could be used as candidate genetic markers for GS SNP chips development; on the other hand, we verified the transcriptional regulation of 22:g.54362761A>G on *SEC13* gene, providing research direction for further function validation tests.

## 1 Introduction

The traits that affect the production efficiency of dairy cows are calving traits, milk production traits, longevity traits and so on (Weller and Ezra 2016; Zhang H. et al., 2021), among which, milk production traits are the most important economic traits in dairy cow breeding, including milk yield, fat yield, protein yield, fat percentage and protein percentage (Spelman et al., 1996). They are quantitative traits, controlled by multiple genes and easily affected by the environment. In addition, dairy cattle breeding is faced with problems such as long generation interval and slow progress (Wiggans et al., 2017). In 2009, developed countries began to widely use genomic selection (GS) for dairy cattle breeding. GS could shorten the generation gap, accelerate the population genetic progress, reduce the breeding costs, and almost double the genetic progress rate (Wiggans et al., 2017). Since the promotion and application of GS in China in 2012, the genetic progress of Chinese Holstein cows has been significantly improved. According to studies, adding functional gene information with greater genetic effects of target traits to single nucleotide polymorphism (SNP) marker data can improve the accuracy of genomic estimated breeding value prediction (Zhang et al., 2014; Zhang et al., 2015; de Las Heras-Saldana et al., 2020). Therefore, many researchers try to find the key genes/loci that have significant impacts on milk production traits through candidate gene analysis, genome-wide association analysis and omics data analysis strategies (Schennink et al., 2009; Canovas et al., 2013; Cui et al., 2014; Liang et al., 2017; Bhat et al., 2020). Studies have shown that the milk production traits of dairy cows can be significantly affected by SNPs in the genes (Jiang et al., 2010; Dux et al., 2018; Han et al., 2019a; Han et al., 2019b; Clancey et al., 2019; Jia et al., 2021; Du et al., 2022; Fu et al., 2022; Ye et al., 2022).

Previously, we used isobaric tag for relative and absolute quantification (iTRAQ) technique to study the proteomes of nine liver tissue samples from three Holstein cows during dry period, early and peak lactations, and found that *SEC13* homolog, nuclear pore and COPII coat complex component (*SEC13*) gene was differentially expressed among different lactations (dry period vs peak lactation: fold change = 1.37, *p*-value = .03134; early lactation vs. peak lactation: fold change = 1.22, *p*-value = .001002) and significantly enriched in the metabolic items and pathways related to milk synthesis, such as citric acid cycle, fatty acid, starch and sucrose metabolism, mTOR and PPAR signal pathways (Xu et al., 2019). *SEC13*, a member of the WD-Repeat protein family, is the core component of the COPII complex in the cytoplasm and nuclear pore complex on the nuclear membrane (Antonny and Schekman 2001; Stagg et al., 2006). Moreover, *SEC13* shuttles between the nucleus and the cytoplasm, acting in the ER-to-Golgi vesicular transport system (Enninga et al., 2003). *SEC13* interacts with *SEC31* to form the outer cage of the COPII vesicle coat needed for the transport of vesicle proteins from the endoplasmic reticulum (ER) to the Golgi matrix (Stagg et al., 2006; Stagg et al., 2008). COPII coat is used to transport newly synthesized proteins, including secretory and transmembrane proteins. As part of the COPII complex, *SEC13* is involved in the process of insulin stimulating glucose uptake by controlling the amount of glucose transporter 4 (GLUT4) in the plasma membrane (Martin et al., 2000; Karylowski et al., 2004; Huang and Czech 2007; Stockli et al., 2011). In addition, we found that an SNP (rs109645852; Chr.22:54428720) 67 kb away from *SEC13* gene (Chr.22:54326136.54361315; Cattle Quantitative Trait Locus Database; https:// www.animalgenome.org/jbrowse/q?data=db%2FbovARS&loc=Chr.22%3A54243801.54530800&tracks=Milk%20composition%20-%20fat%2CGenbank%20annotations&highlight=Chr.22%3A54428718.54428722%20(%22Milk%20fat%20yield%20(daughter%20deviation)%22) was significantly associated with milk fat yield, which was identified in a reported genome-wide association study of Holstein cows (*p* = .0359) (Meredith et al., 2012). Therefore, we inferred that *SEC13* gene might be an important functional gene affecting milk production traits of dairy cows.

In this study, based on the candidate gene *SEC13* for milk production traits of dairy cows, the SNPs of this gene were detected, their genetic associations with milk yield, fat yield, fat percentage, protein yield and protein percentage were analyzed, and whether they could be used in the development of GS SNP chips was evaluated. In addition, we predicted the effect of identified SNPs on transcription factor binding site (TFBS), and verify the effect of SNP at 5′flanking region on the transcriptional activity of *SEC13* gene by dual-luciferase reporter experiment, thus speculating the causal mutation of milk production traits in dairy cows.

## 2 Materials and methods

### 2.1 Animal selection and phenotypic data collection

In this study, 947 cows from 45 Chinese Holstein sire families in Beijing were used as the experimental population. They were distributed in 22 farms of Beijing Shounong Animal Husbandry Development Co., Ltd. (Beijing, China), and raised under the same feeding conditions, with accurate pedigree and Dairy Herd Improvement (DHI) records. We used the phenotypic data of 947 first-lactation cows and 654 second-lactation cows (293 cows only completed the first lactation) for association analysis. The data of the entire lactation period of the parity of each cow was used as the individual milk yield phenotype. The actual total milk yield was multiplied by the corresponding estimated coefficient to get 305-day milk yield. The 305-day milk fat and protein content were calculated by multiplying the 305-day milk yield by the average percentage of milk fat and protein, respectively. The average milk fat and protein percentages were the ratio of total milk fat and protein contents to total milk yield, respectively. Results of descriptive statistics for the milk yield and composition in the two lactations were shown in Supplementary Table S1. Frozen semen and cow blood samples were provided by Beijing Dairy Center (Beijing, China).

### 2.2 Genomic DNA extraction

We extracted genomic DNA from frozen semen of 45 bulls by salt-out procedure, and used a TIANamp Blood DNA Kit (Tiangen, Beijing, China) to extract DNA from the blood of 947 cows. We detected the quantity and quality of extracted DNA samples by a NanoDrop2000 spectrophotometer (Thermo Science, Hudson, NH, United States) and gel electrophoresis respectively.

### 2.3 SNP identification and estimation of linkage disequilibrium

We designed primers with Primer3 (http://bioinfo.ut.ee/primer3-0.4.0/) to amplify all the coding regions and the 2000 bp of 5′and 3′regions of *SEC13* gene. The primers were synthesized by Beijing Genomics Institute (BGI, Beijing, China). The genomic DNA of bull frozen semen was mixed with the same amount, then amplified by PCR (Supplementary Table S2). We used 2% gel electrophoresis to detect whether the PCR amplification products were qualified, and sequenced the qualified PCR amplification products by Sanger sequencing (BGI, Beijing, China). Then we compared the sequenced sequences with the reference sequences (ARS-UCD1.2) on NCBI-BLAST (https://blast.ncbi.nlm. nih. gov/Blast.cgi) to find the potential SNPs. Subsequently, we used Haploview4.2 (Broad Institute of MIT and Harvard, Cambridge, MA, United States) to estimate the degree of linkage disequilibrium (LD) between the identified SNPs. In addition, we genotyped 947 dairy cows using Genotyping by Target Sequencing (GBTS) technology in Boruidi Biotechnology Co., Ltd. (Hebei, China).

### 2.4 Association analyses on milk production traits

We used the SAS 9.4 software (SAS Institute Inc., Cary, NC, United States) to estimate the genetic associations of the SNPs or haplotype blocks with milk production traits, 305-day milk yield, fat yield, fat percentage, protein yield and protein percentage, on first or second lactation with the following animal model: y=µ+HYS+b×M+G+a+e ; where y is the phenotypic value of each trait of each cow; μ is the overall mean; HYS is the fixed effect of farm (1–22: 22 farms), year (1–4: 2012–2015) and season (1: April-May; 2: June-August; 3: September-November and 4: December–March); M is the age of calving as a covariant, b is the regression coefficient of covariant M; G is the genotype or haplotype combination effect; a is the individual random additive genetic effect, the distribution is $N\;\left(0,\;A\delta_{a}^{2}\right)$ , the additive genetic variance is $\delta_{a}^{2}$ ; and e is random residual, the distribution is $N\;\left(0,\;I\delta_{e}^{2}\right)$ , the unit matrix I and the residual variance $\delta_{e}^{2}$ . Bonferroni correction was carried out by multiple tests, the significance level was equal to the original *p*-value divided by the number of genotype or haplotype combinations. We also calculated the additive (a), dominant (d), and substitution (α) effects as follows: $a=\frac{AA-BB}{2}$ ;d = $AB-\frac{AA+BB}{2}$ ; $\alpha =a+d\left(q-p\right)$ ; where, AA, BB, and AB are the least square means of the milk production traits in the corresponding genotypes, p is the frequency of allele A, and q is the frequency of allele B.

### 2.5 Prediction and verification of SNP induced changes in gene transcription activity

We used Jaspar software (http://jaspar.genereg.net/) to predict whether SNP in the 5′flanking region of *SEC13* gene changed the transcription factor binding site (TFBS; relative score≥ .90).

Further, we used the dual-luciferase reporter assay to verify the effect of SNP site that affect the transcription factor binding on gene expression activity. For 22:g.54362761A>G, we synthesized the fragment with SNP site, A or G. The fragment that carried endonuclease sites KpnI and Nhel at ends, respectively, were cloned into pGL4.14 luciferase analysis vector (Promega, Madison, WI, United States). The constructed plasmid was sequenced to confirm the integrity of each insert. The Endo-free Plasmid Maxi Kit (Omega Bio-tek, Inc., Norcross, GA, United States) was used to extract plasmids needed for cell transfection. Human embryonic kidney (HEK) 293T cells were cultured in Dulbecco’s modified Eagle’s medium (Gibco; Thermo Fisher Scientific Inc., MA, United States) supplemented with 10% fetal bovine serum (FBS; Gibco) at 5% CO_2_ and 37°C. The cells were seeded into 24-well plates with 2 × 105–107 cells per well before transfection. The cells were transiently transfected with liposome 2000 (Invitrogen; Thermo Fisher Scientific Inc.). For each well, 500 ng of the constructed plasmid was co-transfected along with 10 ng of pRL-TK Renilla luciferase reporter vector (Promega). 48 h after transfection, the cells were harvested and the luciferase activity was detected by a Dual-Luciferase Reporter Assay System (Promega). The relative fluorescence activity was calculated by the fluorescence activity ratio of firefly and renilla.

## 3 Results

### 3.1 SNPs identification

We totally found four SNPs in *SEC13* gene, one SNP, 22:g.54362761A>G, is located in 5′flanking region, 22:g.54334911G>A in intron and two SNPs, 22:g.54326411T>C and 22:g.54326366C>T, in 3′untranslated region (UTR) (Table 1). Additionally, the genotypic and allelic frequencies of all the identified SNPs were summarized in Table 1.

**TABLE 1:** Detailed information about SNPs identified in SEC13 gene and their genotypic and allelic frequencies. UTR: untranslated region.

SNP name	Rs-ID	Region	Genotype	Genotypic frequency	Allele	Allelic frequency
22:g.54362761A>G	rs135591064	5′flanking region	AA	.623	A	.7856
			AG	.3252	G	.2144
			GG	.0517		
22:g.54334911G>A	rs43599316	intron	AA	.2313	A	.4725
			AG	.4826	G	.5275
			GG	.2862		
22:g.54326411T>C	rs133320599	3′UTR	CC	.7645	C	.8786
			CT	.2281	T	.1214
			TT	.0074		
22:g.54326366C>T	rs208189354	3′UTR	CC	.0591	C	.2592
			CT	.4002	T	.7408
			TT	.5407		

### 3.2 Association analysis between SNP and the five milk production traits

We analyzed the associations between the four SNPs and five milk production traits in dairy cows, including 305-day milk yield, fat yield, protein yield, fat percentage and protein percentage (Table 2). The SNP 22:g.54362761A>G was significantly associated with protein yield (*p* = .036) in the first lactation, milk yield (*p* = .0004), fat yield (*p* < .0001) and protein yield (*p* < .0001) in the second lactation. 22:g.54334911G>A was significantly associated with fat yield (*p* = .0351) in the first lactation, milk yield (*p* = .0033), fat yield (*p* < .0001), protein yield (*p* = .0001) and protein percentage (*p* = .0274) in the second lactation. 22:g.54326411T>C was significantly associated with protein yield (*p* = .0296) in the second lactation. 22:g.54326366C>T was significantly associated with milk yield (*p* = .0173), fat yield (*p* = .0008) and protein yield (*p* = .0028) in the first lactation. Accordingly, the results of additive, dominant and substitution effects for the four SNPs were shown in Supplementary Table S3.

**TABLE 2:** Associations of four SNPs with milk production traits in Chinese Holstein cattle during two lactations.

SNPs	Lactation	Genotype (No.)	Milk yield (kg)	Fat yield (kg)	Fat percentage (%)	Protein yield (kg)	Protein percentage (%)
22:g.54362761A>G	1	AA (590)	10296 ± 62.3329	343.61 ± 2.7953	3.3501 ± .02578	304.34 ± 2.0345a	2.9577 ± .008228
		AG (308)	10389 ± 70.5347	342.64 ± 3.1043	3.3082 ± .02899	308.47 ± 2.2602b	2.968 ± .009488
		GG (49)	10329 ± 121.39	346.76 ± 5.0561	3.3665 ± .04899	305.25 ± 3.6856ab	2.9563 ± .01719
		P	.2343	.6475	.1119	.036	.3926
	2	AA (412)	10715 ± 63.3167Aa	384.74 ± 2.8456Aa	3.6115 ± .0262	316.65 ± 2.0709A	2.9664 ± .008361
		AG (212)	10534 ± 78.0057b	377.71 ± 3.3976Ab	3.6095 ± .03195	309.52 ± 2.4743Ba	2.9603 ± .01061
		GG (30)	10195 ± 152.38Bb	357.97 ± 6.2924B	3.53 ± .06133	297 ± 4.5878Bb	2.9419 ± .02173
		P	.0004	<.0001	.3769	<.0001	.4886
22:g.54334911G>A	1	AA (219)	10257 ± 74.9765	344.44 ± 3.2661ab	3.3598 ± .0307	303.45 ± 2.3785	2.9567 ± .01022
		AG (457)	10302 ± 65.3486	340.75 ± 2.9116a	3.3204 ± .02697	304.93 ± 2.1194	2.962 ± .00871
		GG (271)	10419 ± 72.0751	346.68 ± 3.1562b	3.3477 ± .02957	308.36 ± 2.2982	2.9621 ± .009768
		P	.0621	.0351	.222	.0582	.8314
	2	AA (145)	10451 ± 86.4939Aa	374.52 ± 3.7205Aa	3.6043 ± .03527	307.98 ± 2.7101Aa	2.9625 ± .01191ab
		AG (327)	10644 ± 68.659b	378.51 ± 3.0478Aa	3.5789 ± .0283	312.54 ± 2.2187Aa	2.9519 ± .009178ab
		GG (182)	10772 ± 76.8091Bb	390.83 ± 3.344B	3.6486 ± .03145	319.71 ± 2.4352B	2.981 ± .01047b
		P	.0033	<.0001	.0651	.0001	.0274
22:g.54326411T>C	1	CC (724)	10332 ± 61.5915	342.78 ± 2.7712	3.3323 ± .02551	305.76 ± 2.0168	2.9608 ± .008119
		CT (216)	10289 ± 75.681	345.21 ± 3.2968	3.3589 ± .03099	304.8 ± 2.4008	2.9624 ± .01032
		TT (7)	10803 ± 281.57	356.33 ± 11.4228	3.287 ± .1127	314.18 ± 8.3315	2.8937 ± .04078
		P	.1582	.3467	.4835	.4734	.2365
	2	CC (504)	10624 ± 61.7697	381.29 ± 2.789	3.6076 ± .02561	313.01 ± 2.0296a	2.9623 ± .008109
		CT (145)	10751 ± 86.3478	385.02 ± 3.721	3.6128 ± .03523	318.76 ± 2.7104b	2.9715 ± .01188
		TT (5)	10989 ± 341.35	387.11 ± 13.8933	3.5485 ± .1367	324 ± 10.1328ab	2.9434 ± .04927
		P	.1661	.4657	.8873	.0296	.628
22:g.54326366C>T	1	CC (56)	10417 ± 112.99ab	346.02 ± 4.7173ab	3.3286 ± .04562	306.98 ± 3.4384ab	2.9536 ± .01599
		CT (379)	10236 ± 67.6416a	338.53 ± 2.9983Aa	3.3209 ± .02786	302.32 ± 2.1827Aa	2.955 ± .00905
		TT (512)	10378 ± 64.3329b	346.59 ± 2.8714Bb	3.3522 ± .02657	307.69 ± 2.09Bb	2.9657 ± .008536
		P	.0173	.0008	.3494	.0028	.3602
	2	CC (36)	10807 ± 137.95	381.62 ± 5.7148	3.566 ± .05557	319.77 ± 4.1663	2.9653 ± .01965
		CT (262)	10695 ± 72.6125	382.4 ± 3.2	3.5963 ± .02985	316.3 ± 2.3298	2.9671 ± .009777
		TT (356)	10608 ± 66.2633	381.76 ± 2.9521	3.619 ± .02734	312.29 ± 2.1488	2.9617 ± .008821
		P	.2174	.9696	.5142	.0518	.8565

Note: The number in the table represents the mean ± standard deviation; the number in the bracket represents the number of cows for the corresponding genotype; *p*-value shows the significance for the genetic effects of SNPs; a, b, c within the same column with different superscripts means *p* < .05; and A, B, C within the same column with different superscripts means *p* < .01.

### 3.3 Association between haplotype block and the five milk traits

**FIGURE 1:**
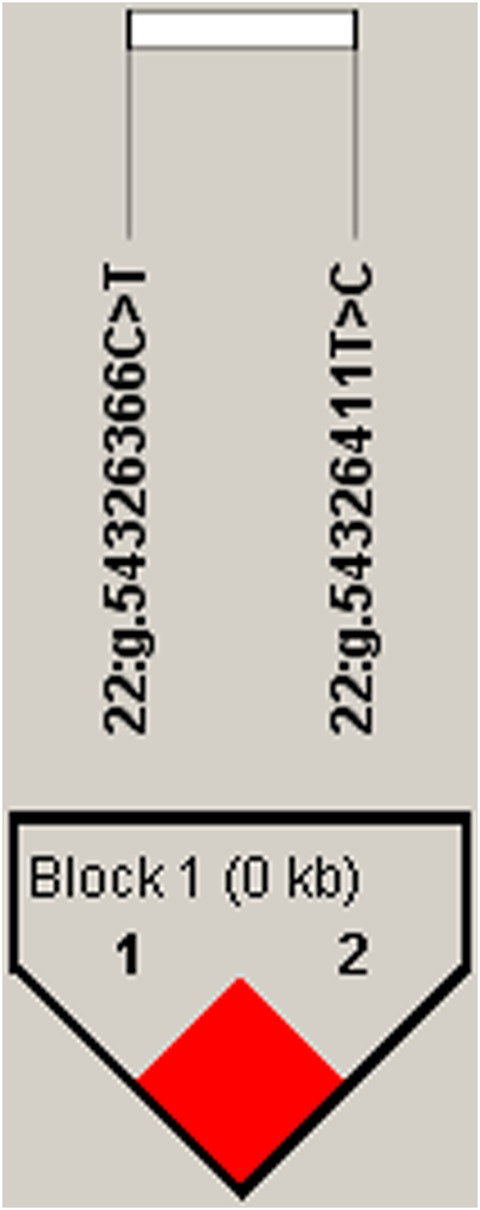
Linkage disequilibrium estimated between SNPs in SEC13 gene. The bright red boxes without numbers indicate complete LD (D′ = 1).

We estimated the degree of LD among four identified SNPs using Haploview4.2, and inferred one haplotype block, including two SNPs, 22:g.54326366C>T and 22:g.54326411T>C, (D' = 1; Figure 1). In the block, the frequencies of H1 (TC), H2 (CC) and H3 (CT) haplotypes were 74.1%, 13.8% and 12.1%, respectively. The haplotype block of *SEC13* was significantly associated with milk yield (*p* = .046), fat yield (*p* = .0001), fat percentage (*p* = .0113), protein yield (*p* = .0056) and protein percentage (*p* = .0373) in the first lactation (Supplementary Table S4).

### 3.4 Effect of 22:g.54362761A>G on gene transcriptional activity

We predicted the TFBS changes for the SNP, 22:g.54362761A>G, in the 5′flanking region of *SEC13* gene by Jaspar software (relative score (RS) ≥.90). The result showed that allele A of 22:g.54362761A>G created binding sites for transcription factors (TFs) GATA5 (RS = .94), GATA3 (RS = .93), HOXD9 (RS = .93), HOXA10 (RS = .91), HOXD13 (RS = .90) and CDX1 (RS = .91; Table 3).

**TABLE 3:** Transcription factor binding sites (TFBSs) prediction for SNP in *SEC13* gene.

SNP name	Allele	Transcription factor	Relative score (RS ≥ .90)	Predicted binding site sequence
22:g.54362761A>G	A	GATA5	.94	CGATAAAA
		GATA3	.93	CGATAAAA
		HOXD9	.93	GCGATAAAAT
		HOXA10	.91	AGCGATAAAAT
		CDX1	.91	GCGATAAAA
		Hoxd13	.90	AGCGATAAAA
	G			

Note: The underlined nucleotides represent the position of the SNP.

**FIGURE 2:**
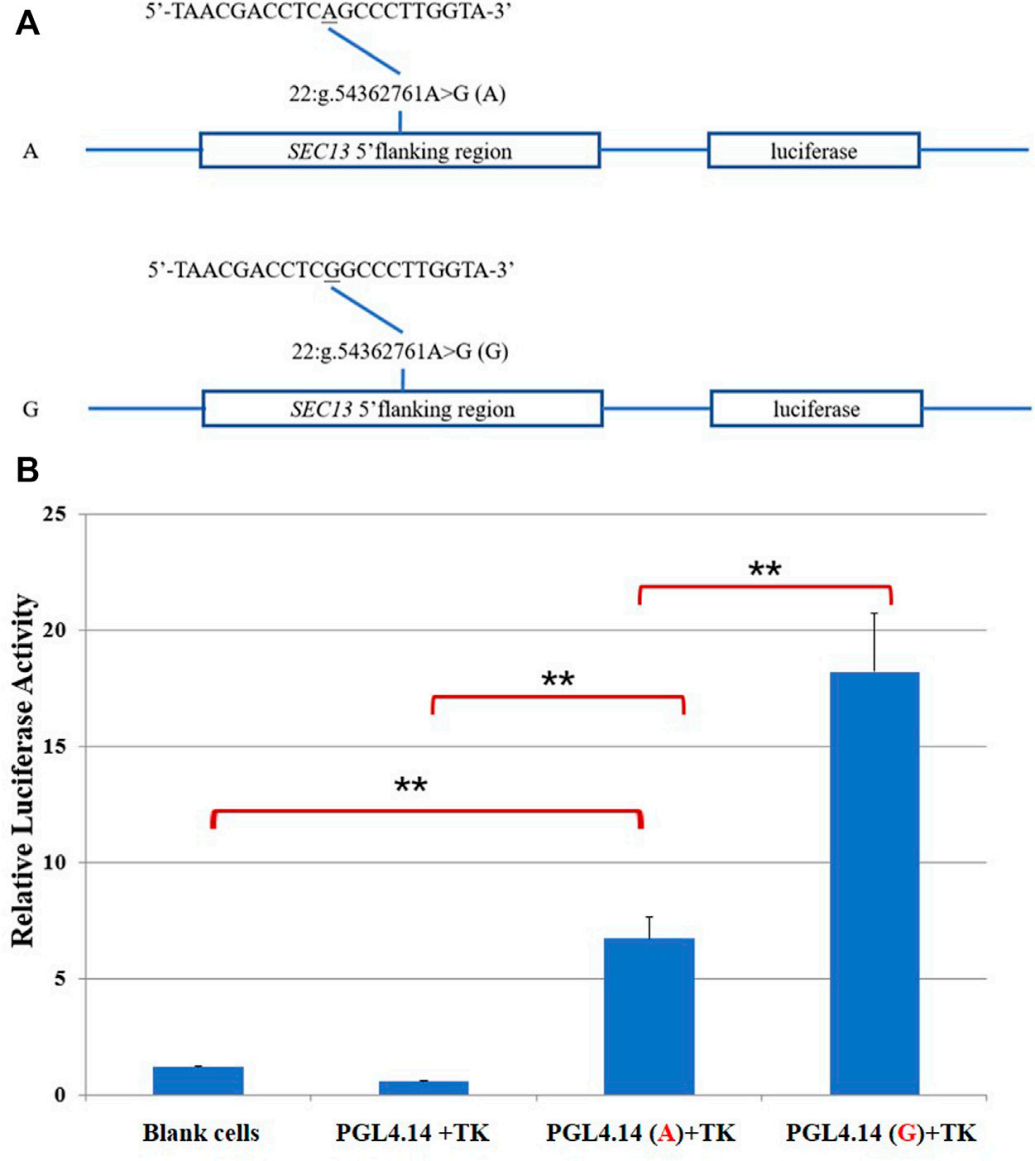
Dual-luciferase activity assay. **(A)**Sketches of recombinant plasmids with 22:g.54362761A>G in SEC13 gene. The underlined nucleotide was the SNP. **(B)** Luciferase activity analysis of the recombinant plasmids in HEK 293T cells. **p < .01.

To further determine whether this SNP changed the transcription activity of *SEC13* gene, we constructed reporter plasmids containing two alleles A and G, respectively (Figure 2A). As shown in Figure 2B, the luciferase activities of the two recombinant plasmids were significantly higher than that of the empty vector (PGL4.14 + TK) and the blank cell controls (*p* < .01), which confirmed that the inserted fragment have transcriptional regulation function. The luciferase activity of G allele (PGL4.14(G)+TK) was significantly higher than that of A allele (PGL4.14(A)+TK; *p* < .01), suggesting that the 22:g.54362761A might inhibit the transcription activity of *SEC13* gene.

## 4 Discussion

Our previous study considered *SEC13* gene to be a candidate to affect milk production traits in dairy cows. In this study, we detected the polymorphisms of *SEC13* gene, and found that there was a significant genetic association between the SNP/haplotype block of the gene and milk production traits. Studies have shown that in the genomic relationship matrices, SNPs can be given different weights to make more accurate and less biased prediction of traits (Tiezzi and Maltecca 2015). Therefore, when adding the SNPs that have significant effects on milk production traits in this study to the commercial SNP chip, we can give different weights to these SNPs according to their effects to improve the accuracy of GS.

Coding sequence (CDS) is a key area for creature survival, many mutations in this region can lead to death, and DNA repair in this region is more efficient than other regions. A studies had shown that the number of SNP in the exon region is less than that in other regions (Frigola et al., 2017), which may be the reason why we didn’t find SNP in the exon region. Transcription factors (TFs) can affect gene expression by combining to TFBSs to regulate the transcription of target genes (Calkhoven and Ab 1996). The SNP located at TFBS may affect the binding of TFs, resulting in differences in gene expression among individuals with different genotypes (Kasowski et al., 2010; McDaniell et al., 2010). In this study, we found that the mutation from allele A to G of 22:g.54362761A>G led to the disappearance of TFBSs for TFs GATA5, GATA3, HOXD9, HOXA10, HOXD13 and CDX1. GATA family regulates cell reprogramming to induce stem cell differentiation and normal function of cells (Shu et al., 2015). GATA5 is a member of GATA family and plays an important role in cardiovascular disease (Shi et al., 2014; Messaoudi et al., 2015). Furthermore, GATA5 is involved in suppressing expression of the reprogramming genes and stemness markers in hepatocellular carcinoma cells (Feng et al., 2020). GATA3 is estimated to be the most highly expressed transcription factor in the differentiated luminal epithelial cells lining the breast ductal structures and plays an important role in mammary gland development (Kouros-Mehr et al., 2006; Kouros-Mehr and Werb 2006). Moreover, GATA3 transcriptionally inhibits Slug expression, thereby inhibits cancer cell proliferation, migration and invasion (Zhang Z. et al., 2021). HOX is a family of many TFs that involved in embryonic development and the maintenance of normal tissues (Mallo and Alonso 2013). HOXD9 is closely related to many kinds of tumors. It can promote tumorigenesis and metastasis by increasing the expression level of other genes in gastric and breast cancer (Zhu et al., 2019; Xiong et al., 2020; Hu et al., 2022). Both HOXA10 and HOXD13 can promote the occurrence and development of various cancers by increasing the expression of genes and activating signal pathways (Chen et al., 2019; Cui et al., 2020; Yin and Guo 2021), nevertheless, both of them can inhibit the expression of genes and suppress the occurrence of prostate cancer (Hatanaka et al., 2019; Xu et al., 2021). CDX1 controls intestinal cell differentiation in the colon and has been shown to directly promote the expression of structural proteins important for epithelial differentiation, including cytokeratin 20 and villin (Chan et al., 2009; Arango et al., 2012). These TFs can activate or inhibit gene expression, and they may interact with each other to promote or inhibit the expression of *SEC13* gene. It has been reported that the *SEC13* gene is close to the quantitative trait locus (QTL) which has been found to have a significant effect on milk fat yield (Meredith et al., 2012). Another study showed that the QTL from the same region showed significant genetic effects on milk protein and fat traits (Bagnato et al., 2008). According to the phenotypic data of milk production traits of different genotypes, it was found that the milk, fat and protein yields of genotype AA was significantly higher than that of genotype GG of 22:g.54362761A>G. Further, the results of dual-luciferase reporter assay showed that when the A mutated to G in 22:g.54362761A>G, the transcriptional activity of *SEC13* gene increased significantly. Therefore, we speculated that the change of *SEC13* gene expression caused by SNP 22:g.54362761A>G may be one of the reasons for the phenotypic change of milk production traits in dairy cows. The specific mechanism of *SEC13* gene on the formation of milk production traits needs further experimental verification. SEC23B, which is also the core component of COPII complex with *SEC13*, mediates the transport of substances from the endoplasmic reticulum to the Golgi complex and plays an important role in professional secretory tissues/cells (Tao et al., 2012). This also provides an important clue for our in-depth research. Further, we can use secretory cell models such as bovine mammary epithelial cells to verify the influence of *SEC13* on the formation of milk production traits.

## 5 Conclusion

This study confirmed the genetic effects of *SEC13* on milk production traits of Chinese Holstein cows. The SNP 22:g.54362761A>G might be a causal mutation site for milk production traits, which might regulate the transcriptional activity of *SEC13* gene by binding transcription factors, the specific mechanism remains to be further verified. This study laid a foundation for the further functional verification of *SEC13* for milk synthesis in dairy cattle, and the significant SNP sites could be used as genetic markers for dairy cattle GS breeding.

## Data Availability

The datasets presented in this study can be found in online repositories. The names of the repository/repositories and accession number(s) can be found in the article/Supplementary Material.

## Supplementary material

SUPPLEMENTARY TABLE S1 Descriptive statistics of phenotypic values for milk production in two lactations.

SUPPLEMENTARY TABLE S2 Primers and procedures for PCR used in SNPs identification of *SEC13* gene.

SUPPLEMENTARY TABLE S3 Additive, dominant and allele substitution effects of four SNPs in *SEC13* gene on milk yield and composition traits in Chinese Holstein cows during two lactations.

SUPPLEMENTARY TABLE S4 Haplotype analysis for *SEC13* gene.
